# Supplementary material for: Light-sensing via hydrogen peroxide and a peroxiredoxin
Source: Nat Commun. 2017 Mar 24;8:14791. doi: 10.1038/ncomms14791 (PMC5376668; doi:10.1038/ncomms14791)
Supplement: Supplementary Information — Supplementary Figures, Supplementary Tables, Supplementary Notes and Supplementary References [file ncomms14791-s1.pdf]

Supplementary Figures

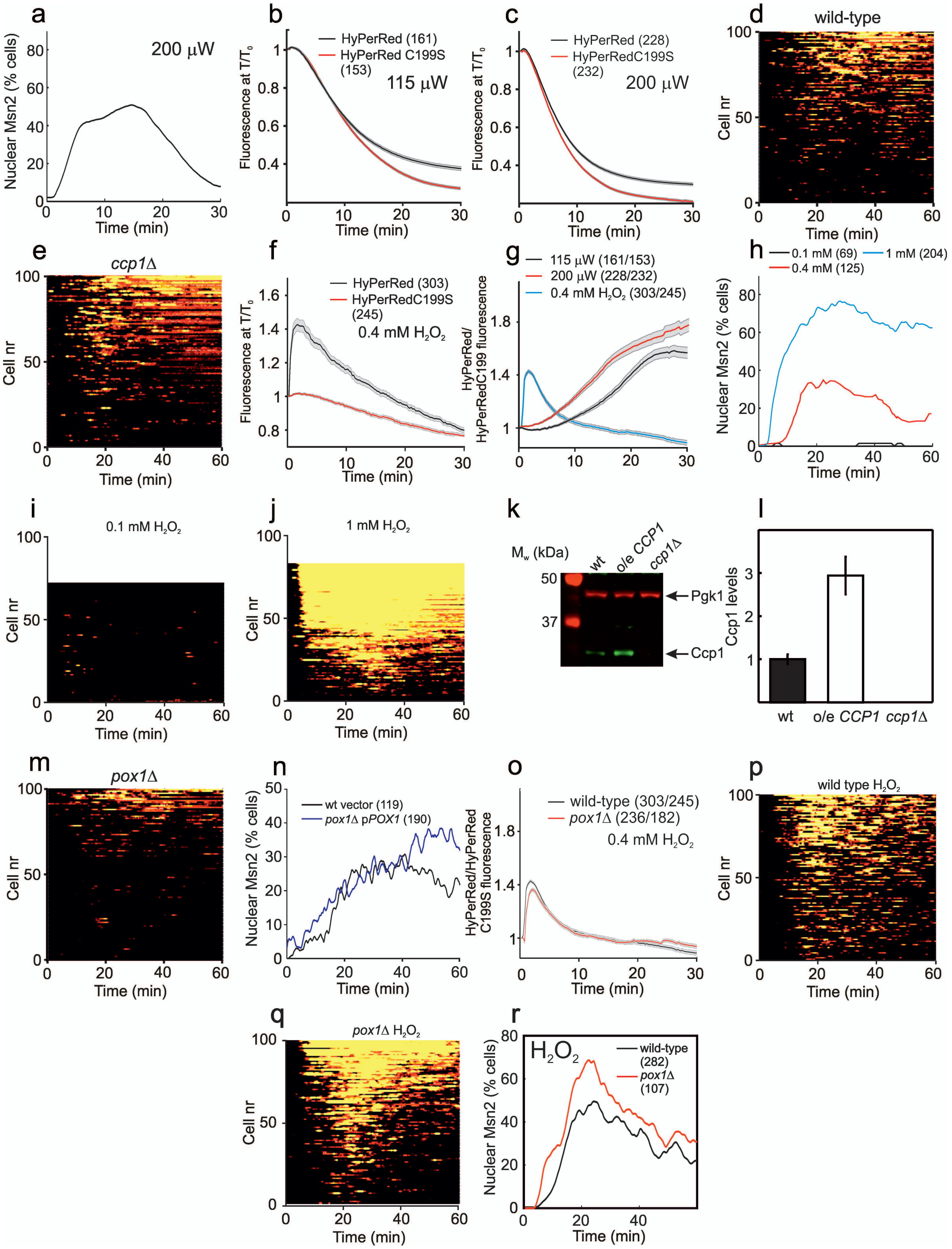

Supplementary Figure 1. H_2O_2 originating in the flavin-containing oxidase Pox1 is necessary and sufficient for light-induced Msn2 nuclear translocation. **a.** Fraction of cells with nuclear localization of Msn2 upon illumination at 200 μW. **b, c, f, g.** HyPerRedSc and HyPerRedScC199S fluorescence following illumination at 115 μW (**b**), 200 μW (**c**) or the addition of 0.4 mM H_2O_2 (**f**). Fluorescence values at the indicated time points were

normalized to the starting values[1]. Error bars indicate SEM. **d-e.** Msn2 nucleocytoplasmic localization trajectories, in individual cells, of the indicated strains following illumination at 115 μW. **g.** Cytosolic H_2O_2, as determined by C199-dependent HyPerRedSc fluorescence, following illumination at 115 μW, 200 μW or the addition of 0.4 mM exogenous H_2O_2. Error bars indicate SEM. **h-j.** Fraction of cells with nuclear localization of Msn2 (**h**) and nuclear localization trajectories in individual wild-type cells (**i-j**) following the addition of the indicated amount of H_2O_2. **k.** Western blot analysis of Ccp1 levels using anti-Ccp1 serum (green). As a loading control, Pgk1 was detected using an anti-Pgk1 antibody (red). **l.** Average levels of Ccp1 normalized to Pgk1 levels in two independent o/e *CCP1* clones. Error bars indicate SD. **n.** Fraction of wild-type vector control cells [p5586[2]] or *pox1Δ* cells transformed with a centromeric *POX1* plasmid [pMOBY-*POX1*[2]] displaying nuclear Msn2 localization following illumination at 115 μW. **o.** Cytosolic H_2O_2, as determined by C199-dependent HyPerRedSc fluorescence, following the addition of 0.4 mM exogenous H_2O_2 to wt and *pox1Δ* cells. Error bars indicate SEM. **p-r.** Nuclear localization trajectories (**p, q**) and fraction of cells with nuclear localization (**r**) of Msn2-GFP in wild-type (**p**) and *pox1Δ* (**q**) cells following H_2O_2 addition (0.4 mM).

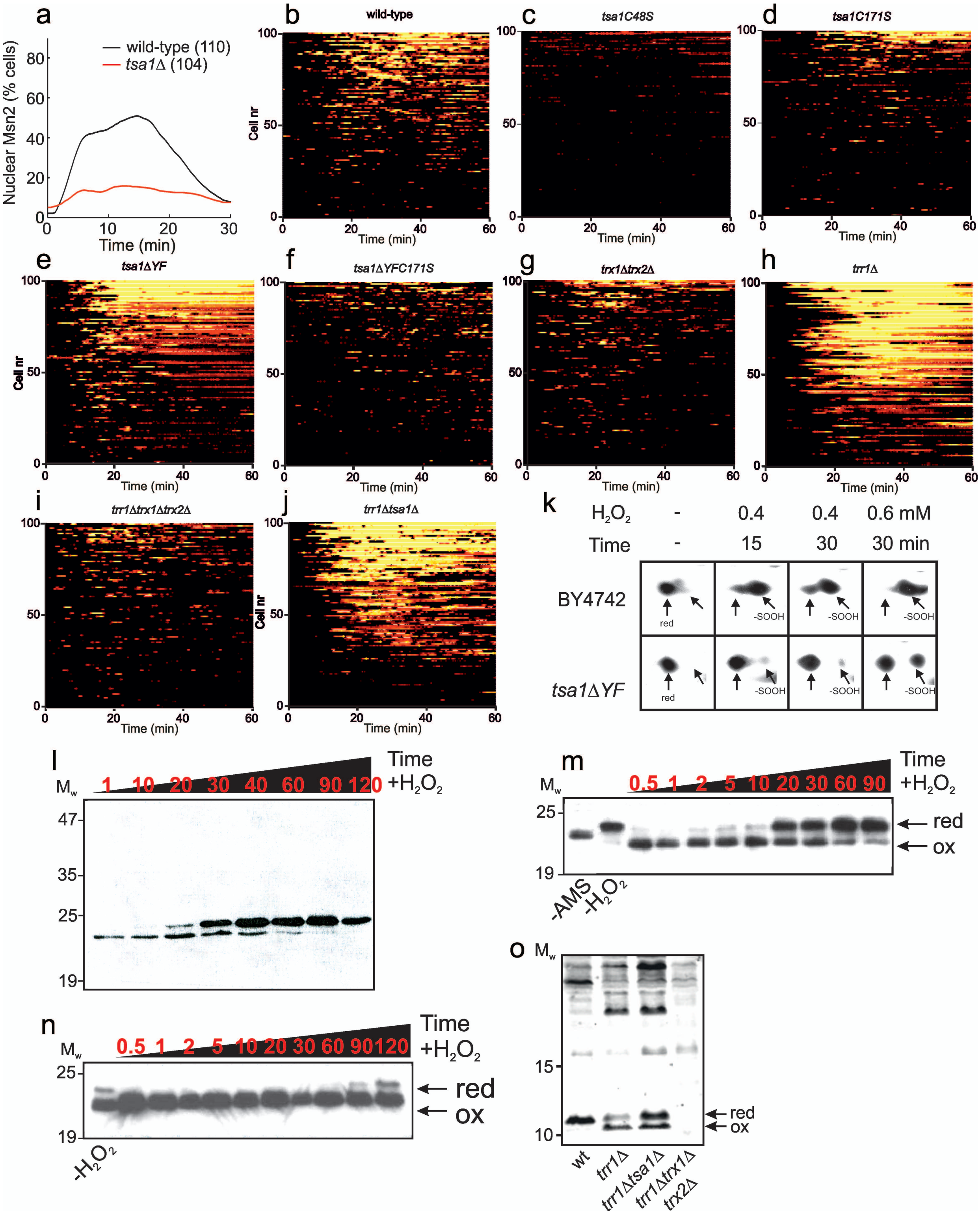

Supplementary Figure 2. The H_2O_2 signal is transduced by the peroxiredoxin Tsa1, which relays the signal to the cytosolic thioredoxins. **a.** Fraction of wt or *tsa1Δ* cells displaying nuclear localization of Msn2 upon illumination at 200 μW. **b-j.** Nucleocytoplasmic localization trajectories in individual cells of the indicated strains following illumination at 115 μW. **k.** 2D-PAGE analysis of Tsa1 hyperoxidation [Tsa1-SOOH[3]] in the wild type (BY4742) or the *tsa1ΔYF* mutant (YMM147) following treatment with H_2O_2 at the indicated concentrations and duration. **l-n.** Trx2 oxidation following treatment or not of wild-type (**l-m**) or *trr1Δ* mutant (**n**)

cells with 0.3 mM (**l,** showing an uncropped blot picture of results in Fig. 2e) or 0.2 mM H_2O_2 (**m**-**n**) for the indicated time, as revealed by redox immunoblot analysis of strains transformed with pRS315-*TRX2*-ProteinA following 4-acetamido-4′-maleimidylstilbene-2,2′-disulfonic acid (AMS) treatment of extracts. AMS slows down the migration of reduced Trx2 by adding 2 × 0.5 kDa. –AMS indicates a sample which was not treated with AMS. **o.** Thioredoxin oxidation in the indicated strains as revealed by redox immunoblot analysis using an anti-thioredoxin antibody and AMS. An uncropped blot picture of results presented in Fig. 2h is shown.

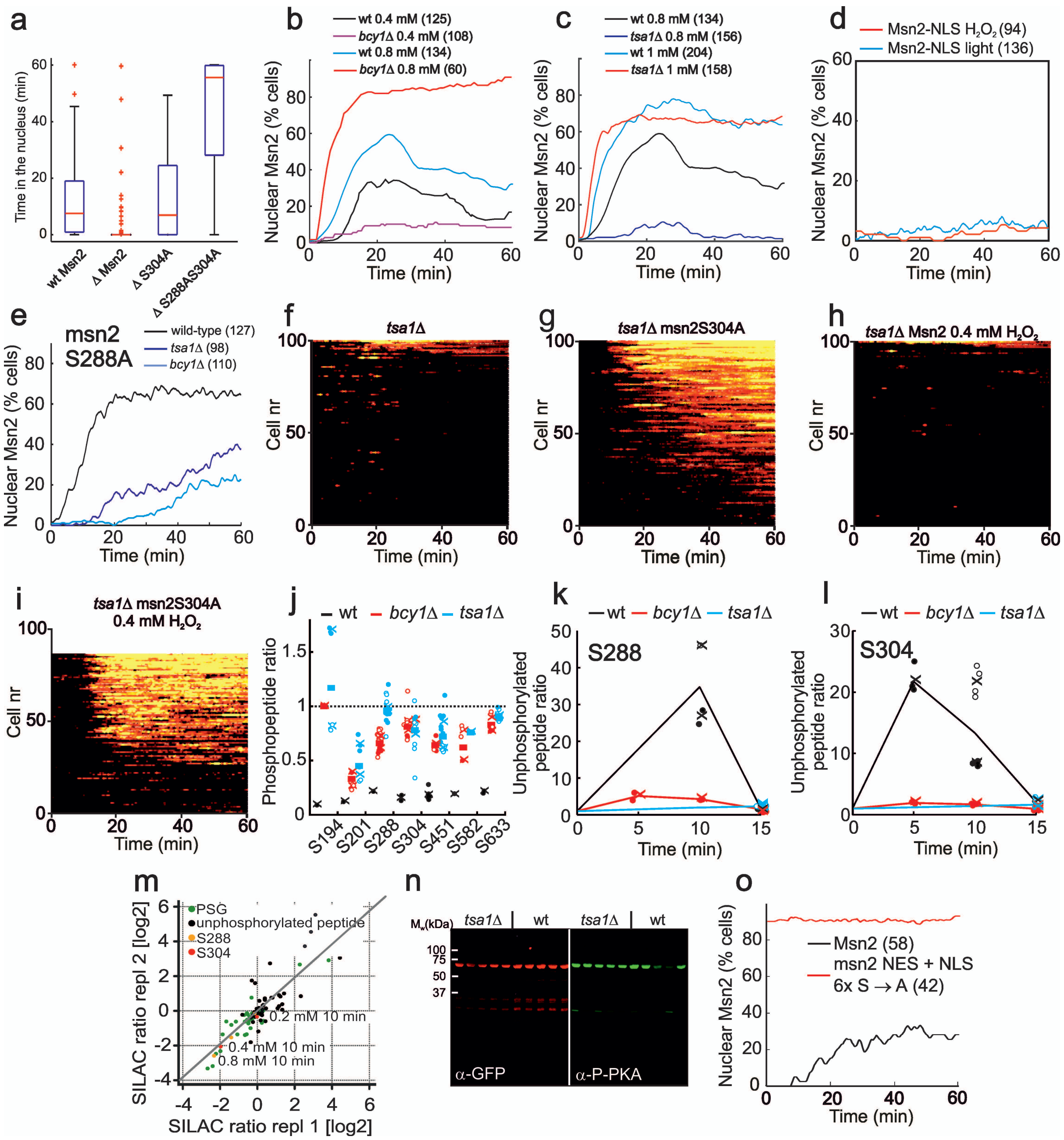

Supplementary Figure 3. Tsa1 controls Msn2 nuclear translocation upon illumination and physiologically relevant H_2O_2 levels by reducing Msn2 NES phosphorylation. **a.** Time Msn2 spent in the nucleus in wt or *bcy1Δ* cells (Δ) expressing the indicated Msn2 alleles upon 0.4 mM H_2O_2. **b, c.** Fraction of the indicated cells displaying nuclear localization upon the addition of H_2O_2. **d-e.** Fraction of cells expressing the Msn2NLS (aa 576-704) fused to an ectopic NES (PKI-NES) and GFP[36] (**d**) or the indicated strains (**e**) displaying nuclear localization following illumination at 115 µW. **f-i.** Nucleocytoplasmic localization trajectories following the illumination of *tsa1Δ* cells expressing wt Msn2 (**f, h**) or an *msn2S304A* mutant (**g, i**) at 115 µW (**f, g**) or the addition of 0.4 mM H_2O_2 (**h, i**). **j.** SILAC-based mass spectrometric quantification of Msn2 phosphopeptides spanning PKA-regulated serines[4] in the indicated strains upon 0.4 mM H_2O_2 for 15 min. Bars represent average ratios of H_2O_2-treated/untreated peptide quantities in two biological replicates (each indicated by a cross). Closed and open circles denote individual peptide ratios quantified in biological replicates 1 and 2, respectively (see Methods and Supplementary Data 1-2). **k, l.** SILAC-based mass

spectrometric quantification of unphosphorylated Msn2 peptides spanning the indicated PKA-regulated NES serines upon 0.4 mM H_2O_2 for the indicated time to the indicated cells. Unmodified peptides spanning the sequence 287-302 (FSDVITNQFPSMTNSR) were used to estimate unphosphorylated S288 ratios, whereas unmodified NSISHSLDLWNHPK (303–316) was used to estimate unphosphorylated S304 ratios (Supplementary Data 1–2). Lines represent average ratios of H_2O_2-treated/untreated quantities in two biological replicates (each indicated by a cross). Closed and open circles denote individual peptide ratios quantified in biological replicates 1 and 2, respectively (see Methods and Supplementary Data 1-2). **m.** Scatter plot of the quantitative distribution of phosphorylation site groups (PSG) and unphosphorylated peptides between replicates 1 and 2 of key experiments. The distribution of phospho-serines 288 (yellow) and 304 (red) upon 0.2 - 0.8mM H_2O_2, for 10 minutes are highlighted. The grey line indicates correlation. **n.** Msn2 NES phosphorylation determined by immunoblot analysis. A representative uncropped blot picture of results presented in Fig. 3g-h is shown. **o.** Fraction of cells displaying nuclear localization of Msn2 or a mutant Msn2 (msn2 NES + NLS 6 S →A) following illumination at 115 μW. To circumvent slow growth due to constitutive nuclear localization[5], Msn2 was conditionally expressed from the *GAL1* promoter in plasmids pWR386 (Msn2) or pKP7 (msn2 NES + NLS 6 S →A) by a hybrid transcription factor (ADGEV) activated by β-estradiol[6] (plasmid pRS316-ADGEV).

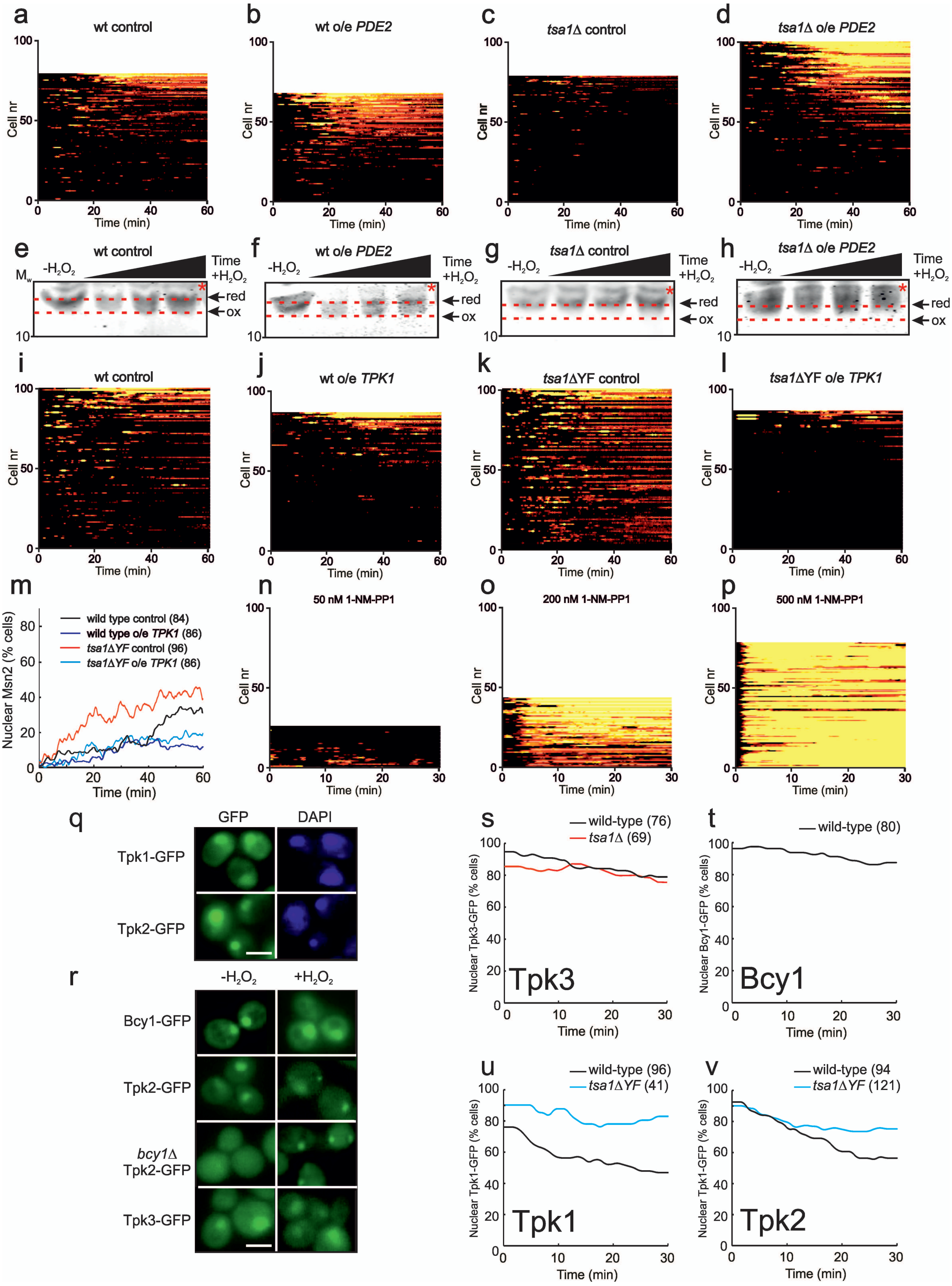

Supplementary Figure 4. Reduced PKA activity is sufficient for Msn2 oscillation and nuclear localization whereas response delay requires Tsa1-mediated inhibition of the nuclear retention of PKA catalytic subunits. **a-d, i-l.** Nucleocytoplasmic localization trajectories following the illumination of the indicated strains transformed with Msn2-GFP and vector controls (**a, c** plasmid yEP352; **i, k** plasmid yEPlac195) or plasmids overexpressing (o/e) *PDE2* (**b, d**) or the

PKA catalytic subunit *TPK1* (**j, l**) at a light intensity of 115 μW. **e-h.** Thioredoxin oxidation in the indicated strains following the addition of 0.3 mM H_2O_2. Samples for protein extraction were withdrawn before and at time-points 1, 10 and 20 minutes following the addition of H_2O_2. The red star (*) indicates an unspecific band recognized by the anti-Trx1/2 antibody[7]. **m.** Fraction of cells of the indicated strains displaying nuclear localization of Msn2 at the indicated time points following illumination. **n-p.** Msn2 nucleocytoplasmic localization trajectories in a strain carrying analog-sensitive PKA catalytic subunits [*tpk1M164Gtpk2M147Gtpk3M165G*[8]] treated with an inhibitory substrate analog (1-NM-PP1) at the indicated concentration. **q-r**. Localization of the indicated PKA subunit GFP fusions in the absence of H_2O_2 (**q, r**) or 12 minutes following the addition of 0.4 mM H_2O_2 (**r**). Nuclear localization of Tpk1-GFP and Tpk2-GFP was confirmed by DAPI staining (**q**). Scale bar, 5 μM. **s-v.** Quantitative time-lapse analysis of the proportion of cells expressing the indicated PKA subunits tagged by GFP localized in the nucleus following the addition of 0.4 mM H_2O_2.

Supplementary Table 1. Strains used in this study.

Strain	Relevant genotype, as indicated in the text	Genotype	Reference or source
YE0033	Nup49-mCherry	BY4741 *NUP49-mCherry*::*hphNT*	Frederik Eisele
BY4742	wild type	MAT alpha *his3Δ1 leu2Δ0 lys2Δ0 ura3Δ0*	[9]
	trp1Δ	BY4742 *trp1Δ::kanMX4*	Research Genetics[10]
	ccp1Δ	BY4742 *ccp1Δ::kanMX4*	Research Genetics[10]
	pox1Δ	BY4742 *pox1Δ::kanMX4*	Research Genetics[10]
YMM114	*tsa1Δ*	BY4742 *tsa1Δ::natMX4*	[3]
YMM141	*trr1Δ*	BY4742 *trr1Δ::kanMX4*	This study
YMM142	*trr1Δ tsa1Δ*	BY4742 *trr1Δ::kanMX4 tsa1Δ::natMX4*	This study
YMM143	*trx1Δ trx2Δ*	BY4742 *trx1Δ::hphMX4 trx2Δ::natMX4*	This study
YMM144	*trr1Δtrx1Δ trx2Δ*	BY4742 *trr1Δ::kanMX4 trx1Δ::hphMX4 trx2Δ::natMX4*	This study
YMM145	*tsa1C48S*	BY4742 *tsa1C48S*	This study
YMM146	*tsa1C171S*	BY4742 *tsa1C171S*	This study
YMM147	*tsa1YFΔ*	BY4742 *tsa1(1-184)*	This study
YMM148	*tsa1C171S YFΔ*	BY4742 *tsa1(1-184)C171S*	This study
Y252	wild-type	YPH98	[11]
YMT6	*ahp1Δ*	YPH98 *ahp1Δ::trp1*	[12]
YMT7	*tsa1Δ*	YPH98 *tsa1Δ::trp1*	[12]
	ahp1Δ tsa1Δ	YPH98 *ahp1Δ::kanMX4 tsa1Δ::trp1*	Michel B. Toledano
	trr1Δ	YPH98 *trr1Δ::kanMX4*	[13]
	trr1Δ trx1Δ trx2Δ	YPH98 *trr1Δ::kanMX4 trx1Δ ::URA3 trx2Δ::kanMX4*	[13]
WR175	BY SILAC Msn2-HTBeaq	BY4741 *lys1Δ::kanMX4*	This study

		arg4Δ::kanMX4 Msn2-HTBeaq::hphMX	
YMM149	BY SILAC Msn2-HTBeaq *tsa1Δ*	Mat a, *met15Δ0*, lys^{-}, *arg4Δ::kanMX4 Msn2-HTBeaq::hphMX tsa1Δ::natMX4*	This study
YMM150	BY SILAC Msn2-HTBeaq *bcy1Δ*	Mat a *met15Δ0 lys1Δ::kanMX4 arg4Δ::kanMX4 Msn2-HTBeaq::hphMX bcy1Δ::natMX4*	This study
YTpkCMx3	*tpk1M164G tpk2M147G tpk3M165G*	BY4742 *tpk1M164G tpk2M147G tpk3M165G*	This study
	bcy1Δ	BY4742 *bcy1Δ::kanMX4*	Joakim Norbeck
BY4741	*TPK1-GFP*	Mat a *his3Δ1 leu2Δ0 met15Δ0 ura3Δ0 TPK1-GFP::HIS3*	Research Genetics[14]
YMM160	*TPK1-GFP tsa1Δ*	Mat a *his3Δ1 leu2Δ0 met15Δ0 ura3Δ0 TPK1-GFP::HIS3 tsa1Δ::kanMX4*	This study
YMM161	*TPK1-GFP tsa1C48S*	Mat a *his3Δ1 leu2Δ0 met15Δ0 ura3Δ0 TPK1-GFP::HIS3 tsa1C48S*	This study
YMM162	*TPK1-GFP tsa1YFΔ*	Mat a *his3Δ1 leu2Δ0 met15Δ0 ura3Δ0 TPK1-GFP::HIS3 tsa1(1-184)*	This study
YMM163	*TPK1-GFP bcy1Δ*	Mat a *his3Δ1 leu2Δ0 met15Δ0 ura3Δ0 TPK1-GFP::HIS3 bcy1Δ::kanMX4*	This study
BY4741	*TPK2-GFP*	Mat a *his3Δ1 leu2Δ0 met15Δ0 ura3Δ0 TPK2-GFP::HIS3*	Research Genetics[14]
YMM164	*TPK2-GFP tsa1Δ*	Mat a *his3Δ1 leu2Δ0 met15Δ0 ura3Δ0 TPK2-GFP::HIS3 tsa1Δ::kanMX4*	This study
YMM165	*TPK2-GFP tsa1C48S*	Mat a *his3Δ1 leu2Δ0 met15Δ0 ura3Δ0 TPK2-GFP::HIS3 tsa1C48S*	This study
YMM166	*TPK2-GFP tsa1YFΔ*	Mat a *his3Δ1 leu2Δ0 met15Δ0 ura3Δ0*	This study

		TPK2-GFP::HIS3 tsa1(1-184)	
YMM167	*TPK2-GFP bcy1Δ*	Mat a *his3Δ1 leu2Δ0 met15Δ0 ura3Δ0 TPK2-GFP::HIS3 bcy1Δ::kanMX4*	This study
BY4741	*BCY1-GFP*	Mat a *his3Δ1 leu2Δ0 met15Δ0 ura3Δ0 BCY1-GFP::HIS3*	Research Genetics[14]
BY4741	*TPK3-GFP*	Mat a *his3Δ1 leu2Δ0 met15Δ0 ura3Δ0 TPK3-GFP::HIS3*	Research Genetics[14]
YMM168	*TPK3-GFP tsa1Δ*	Mat a *his3Δ1 leu2Δ0 met15Δ0 ura3Δ0 TPK3-GFP::HIS3 tsa1Δ::kanMX4*	This study

Supplementary Table 2. Plasmids used in this study.

Plasmid	***Replicon***	***Ectopic promoter***	***Gene***	***Selection marker***	Reference
pAMG	CEN/ARS	*ADH1*	*MSN2-GFP*	*LEU2*	[8]
pHyPerRed	CEN/ARS	*GPD (TDH3)*	*HyPerRedSc*	*URA3*	This study
pHyPerRed C199S	CEN/ARS	*GPD (TDH3)*	*HyPerRedScC199S*	*URA3*	This study
pYX132	CEN/ARS	*TPI1*	-	*TRP1*	
pYX132-*CCP1*	CEN/ARS	*TPI1*	*CCP1*	*TRP1*	[15]
p5586	CEN/ARS	-	-	*URA3*	MOBY empty vector[2]
pMOBY-*POX1*	CEN/ARS	-	*POX1*	*URA3*	[2]
pRS315-*TRX2-ProteinA*	CEN/ARS	-	*TRX2-ProteinA*	*LEU2*	This study
pAMG9	CEN/ARS	*ADH1*	*msn2(267-567)-GFP*	*LEU2*	[16]
pHR1668	CEN/ARS	*ADH1*	*msn2S288A-GFP*	*LEU2*	This study
pWR76	CEN/ARS	*ADH1*	*msn2S304A-GFP*	*LEU2*	This study
pWR78	CEN/ARS	*ADH1*	*msn2S288AS304A-GFP*	*LEU2*	This study
pWR386	CEN/ARS	*GAL1*	*GST-MSN2-GFP*	*LEU2*	This study
pKP7	CEN/ARS	*GAL1*	*GST-msn2S288AS304AS582AS620AS625AS633A-GFP*	*LEU2*	This study

pRS316-ADGEV	CEN/ARS	*ADH1*	Gal4(1-93)-hER(282-516)-VP16(424-490)	*URA3*	Peter Pryciak
yEPlac195	2μ	-	-	*URA3*	[17]
pXP2	2μ	-	*TPK1*	*URA3*	[18]
yEP352	2μ	-	-	*URA3*	[19]
pGR103	2μ	-	*PDE2*	*URA3*	[20]

Supplementary Note 1

Reproducibility and statistical analyses

All experiments in this study were repeated at least three times to confirm reproducibility (biological replicates). Results of statistical analyses performed on data can be found below.

Fig. 1d: Responses in mutant cells differ significantly from responses in the wt ($p < 0.05$) at 90% (*ccp1Δ*) or 85% (*pox1Δ*) of the 685 time points sampled (10-60 min), respectively. The number of cells sampled were 239 (wt), 182 (*ccp1Δ*) and 96 (*pox1Δ*). **e.** HyPerRed fluorescence following illumination at 115 and 200 μW differs significantly from the HyPerRedC199S fluorescence acquired at the same light intensities in 11% and 34% of the 120 time-points analyzed, respectively. For both light intensities all significant time-points were between 20 and 30 min. **g.** The response in cells overproducing Ccp1 differs significantly from control cells in 66% of the 685 time-points sampled (10-60 min). The response in wt control cells (carrying plasmid pYX132) does not differ significantly from the wt without the plasmid at any of the 685 time-points assayed. **h.** HyPerRed fluorescence curves following illumination at 115 and 200 μW in *pox1Δ* cells differ significantly from HyPerRedC199S fluorescence in none of the 120 time-points analyzed. HyPerRed fluorescence in wt cells differ significantly from that of *pox1Δ* cells in 13% (115 μW) and 35% (200 μW) of the 120 time-points analyzed.

Fig. 2c, d: Responses in mutant cells differ significantly from the wild type ($p < 0.05$) at 94% (*tsa1Δ*), 90% (*tsa1C48S*), 53% (*tsa1C171S*), 100% (*tsa1ΔYF*), 67% (*tsa1ΔYFC171S*), 79% (*trr1Δ*), and 66% (*trx1Δtrx2Δ*) of the 685 time points (10-60 min). In contrast, *tsa1Δ* vs *tsa1C48S*, *tsa1C171S* vs *tsa1ΔYFC171S,* and *trx1Δtrx2Δ* vs *trr1Δtrx1Δtrx2Δ* differ significantly only at 4%, 10%, and 6% of the time points, respectively. **g.** Responses in mutant cells differ significantly from the wild type ($p < 0.05$) at 79% (*trr1Δ*) and 66% (*trx1Δtrx2Δ*) of the 685 time points (10-60 min). **i.** Responses in *trr1Δtsa1Δ* cells differ significantly from *tsa1Δ* cells in 83% of the time-points and from *trr1Δ* cells in 76% of the time-points (10-60 min).

Fig. 3a. Responses in mutant cells expressing Msn2 differ significantly from the wt ($p < 0.05$) at 96% (*bcy1Δ*), 87% (*pde2Δ*), and 94% (*tsa1Δ*) of the 685 time points (10-60 min). **b.** Responses in cells expressing *msn2S304A* differ from the wild-type at 57% (*tsa1Δ*) and 100% (*bcy1Δ*) of the 685 time points (10-60 min). Responses in *tsa1Δ* and *bcy1Δ* cells expressing *msn2S304A* differ significantly from control *tsa1Δ* and *bcy1Δ* cells in 100% and 73% of the 685 time-points (10-60 min), respectively. **c.** Cells expressing *msn2S288AS304A* show significant difference from the wild-type in none of the time points (*pde2Δ*) or in only 3% (*tsa1Δ*) or 13% (*bcy1Δ*) of the 685 time points (10-60 min). The Msn2 nuclear localization in *bcy1*Δ *msn2S288AS304A* cells is, however, significantly different from the wild-type in 100% of the 137 early time-points (0-10 min). **h.** Differences in Msn2 NES phosphorylation between

strains are statistically significant 5 and 10 min after H_2O_2 addition ($n = 3$, $p = 3.3 \times 10^{-4}$ and 6.0×10^{-4}, respectively, in two sided Student's T-tests).

Fig. 4a. *tsa1Δ* o/e *PDE2* cells show a significant difference from *tsa1Δ* control cells at 99% of the 685 time points but from wt o/e *PDE2* cells at only 49% of the 685 time points (10-60 min). **b.** The total time Msn2 spent in the nucleus of wt control cells differs significantly from that of *tsa1ΔYF* control cells ($p = 2.0 \times 10^{-6}$) and wt o/e *TPK1* cells ($p = 1.0 \times 10^{-8}$), whereas there is no significant difference between wt and *tsa1ΔYF* o/e *TPK1* cells ($p = 0.08$). The number of cells analyzed were wt control (84), wt o/e *TPK1* (86), *tsa1ΔYF* control (96) and *tsa1ΔYF* o/e TPK1 (86). **c.** Responses to the inhibitor show a statistically significant difference ($p < 0.05$) at 98% (50 vs 100 nM) or 100% of the time points sampled (10-60 min). **e.** Msn2 response times for cells illuminated at a light intensity of 115 μW (n=239) are not significantly different from cells treated with 0.4 mM H_2O_2 (n=125, p=0.64) whereas they are significantly different from cells treated with 100 nM 1-NM-PP1 (n=43, $p < 0.0001$). **g.** Tpk1-GFP localization in *tsa1Δ* and *tsa1C48S* mutant cells differ significantly from the wild type ($p < 0.05$) in 100% of the 30 time-points sampled, but from each other in none of the time-points. The Tpk1-GFP response in *bcy1Δ* differs from the wt in 58% of the 30 time-points sampled (1-18 min). **h.** Tpk2-GFP responses in *tsa1Δ* , *tsa1C48S* and *bcy1Δ* mutant cells differ significantly from the wild type ($p < 0.05$) in 77% (*tsa1Δ* , 8-30 min), 84% (*tsa1C48S*, 6-30 min) and 84% (*bcy1Δ*, 0-26 min) of the 31 time-points sampled. **i.** Wt and *bcy1Δ* response times in cells expressing *msn2S288AS304A* are significantly different (p=1.8 x10^{-4}).

Supplementary Fig. 1a. The Msn2 response in wt cells illuminated at 200 μW differs significantly from that of wt cells illuminated at 115 μW in 67% of the 80 time-points measured 10-60 min. **f.** HyPerRed fluorescence is significantly different from HyPerRedC199S fluorescence at 79% of the 120 time-points analyzed following the addition of 0.4 mM H_2O_2. **h.** The Msn2 response of wt cells to 1 mM H_2O_2 differs significantly from that to 0.4 mM H_2O_2 in 100% of the 100 time-points assayed 10-60 min. **n.** Responses in wt vector and *pox1Δ* p*POX1* cells show a statistically significant difference ($p < 0.05$) at only 25% of the 685 time points (10-60 min). **o.** HyPerRedSc fluorescence in wt and *pox1Δ* cells following the addition of 0.4 mM H_2O_2 do not differ significantly in any of the 120 time-points assayed. **r.** Responses in wt and *pox1Δ* to 0.4 mM H_2O_2 show a statistically significant difference ($p < 0.05$) at 96% of the time points.

Supplementary Fig. 2a. The Msn2 responses in wt and *tsa1Δ* cells upon illumination at 200 μW differs significantly in 67% of the 80 time-points assayed 10-30 min.

Supplementary Fig. 3a. The time spent in the nucleus in *bcy1Δ* cells expressing *MSN2* differs significantly from the wt ($p = 7.6$ x10^{-8}) and from *bcy1Δ* cells expressing *msn2S304A* and *msn2S288AS304A* at p=1.3 x 10^{-16} and 2.1 x 10^{-23}, respectively. The number of cells sampled were 239 (wt Msn2), 108 (*bcy1Δ* Msn2), 134 (*bcy1Δ msn2S304A*) and 60 (*bcy1Δ msn2S288AS304A*). **b.** The response in *bcy1Δ* cells at 0.4 mM H_2O_2 differs significantly from that of the wt ($p < 0.05$) at 95% of the time points and at 0.8 mM H_2O_2 at 100% of the 100 time-points 10-60 min. **c.** The response in *tsa1Δ* cells at 0.8 mM H_2O_2 differs significantly from that of the wt ($p < 0.05$) in 100% of the time points and at 1 mM H_2O_2 in 74% of the 100 time-points 10-60 min. **e.** Responses in wt and mutant cells expressing *msn2S288A* differ significantly at 100% of the 685 time points (10-60 min). Responses in *bcy1Δ* and *tsa1Δ* cells expressing *msn2S288A* differ significantly from *bcy1Δ* and *tsa1Δ* control cells in 18% and 72 % of the 685 time-points (10-60 min). **h-i.** The fraction of *tsa1Δ* cells expressing *msn2S304A*

differs significantly from control cells in 100% of the 685 time-points analyzed (10-60 min). The number of cells sampled were 168 (*tsa1Δ MSN2*) and 86 (*tsa1Δ msn2S304A*). **m.** The correlation coefficient between Msn2 phosphorylation measured in two biological replicates using SILAC-based mass-spectrometric phosphoproteomics is 0.873. **o.** The response in the msn2 NES + NLS 6 x S → A mutant is significantly different from the wt Msn2 ($p<0.05$) in 100% of the 685 time-points.

Supplementary Fig. 4m. Msn2 nuclear localization in wild-type control cells show a statistically significant difference from wt o/e *TPK1* cells ($p < 0.05$) at 85% of the time points and from *tsa1ΔYF* control cells at 54% of the time points, whereas wt o/e *TPK1* and *tsa1ΔYF* o/e *TPK1* cells show a significant difference at only 5% of the 685 time points (10-60 min). **s.** Tpk3-GFP nuclear localization only differs significantly ($p<0.05$) in wt and *tsa1Δ* cells in 30% of the 30 time-points sampled. **t.** Bcy1-GFP nuclear localization was measured in 80 cells. **u.** Tpk1-GFP nuclear localization differs significantly ($p<0.05$) in wt and *tsa1YFΔ* cells in 100% of the 30 time-points sampled. **v.** Tpk2-GFP nuclear localization differs significantly ($p<0.05$) in wt and *tsa1YFΔ* cells in 83% of the 30 time-points sampled.

Supplementary references

1 Ermakova, Y. G. *et al.* Red fluorescent genetically encoded indicator for intracellular hydrogen peroxide. *Nature communications* **5**, 5222 (2014).

2 Ho, C. H. *et al.* A molecular barcoded yeast ORF library enables mode-of-action analysis of bioactive compounds. *Nat Biotechnol* **27**, 369-377 (2009).

3 Molin, M. *et al.* Life span extension and H_2O_2-resistance elicited by caloric restriction require the peroxiredoxin Tsa1 in *Saccharomyces cerevisiae*. *Mol Cell* **43**, 823-833 (2011).

4 Reiter, W. *et al.* Yeast protein phosphatase 2A-Cdc55 regulates the transcriptional response to hyperosmolarity stress by regulating Msn2 and Msn4 chromatin recruitment. *Mol Cell Biol* **33**, 1057-1072 (2013).

5 Durchschlag, E., Reiter, W., Ammerer, G. & Schuller, C. Nuclear localization destabilizes the stress-regulated transcription factor Msn2. *J Biol Chem* **279**, 55425-55432 (2004).

6 Takahashi, S. & Pryciak, P. M. Membrane localization of scaffold proteins promotes graded signaling in the yeast MAP kinase cascade. *Curr Biol* **18**, 1184-1191 (2008).

7 Kumar, C. *et al.* Glutathione revisited: a vital function in iron metabolism and ancillary role in thiol-redox control. *EMBO J* **30**, 2044-2056 (2011).

8 Bishop, A. C. *et al.* A chemical switch for inhibitor-sensitive alleles of any protein kinase. *Nature* **407**, 395-401 (2000).

9 Brachmann, C. B. *et al.* Designer deletion strains derived from *Saccharomyces cerevisiae* S288C: a useful set of strains and plasmids for PCR-mediated gene disruption and other applications. *Yeast* **14**, 115-132 (1998).

10 Giaever, G. *et al.* Functional profiling of the *Saccharomyces cerevisiae* genome. *Nature* **418**, 387-391 (2002).

11 Sikorski, R. S. & Hieter, P. A system of shuttle vectors and yeast host strains designed for efficient manipulation of DNA in *Saccharomyces cerevisiae*. *Genetics* **122**, 19-27 (1989).

12 Lee, J., Spector, D., Godon, C., Labarre, J. & Toledano, M. B. A new antioxidant with alkyl hydroperoxide defense properties in yeast. *J Biol Chem* **274**, 4537-4544 (1999).

13 Le Moan, N., Clement, G., Le Maout, S., Tacnet, F. & Toledano, M. B. The *Saccharomyces cerevisiae* proteome of oxidized protein thiols: contrasted functions for the thioredoxin and glutathione pathways. *J Biol Chem* **281**, 10420-10430 (2006).

14 Huh, W. K. *et al.* Global analysis of protein localization in budding yeast. *Nature* **425**, 686-691 (2003).

15 Tatsuta, T., Augustin, S., Nolden, M., Friedrichs, B. & Langer, T. m-AAA protease-driven membrane dislocation allows intramembrane cleavage by rhomboid in mitochondria. *EMBO J* **26**, 325-335 (2007).

16 Gorner, W. *et al.* Nuclear localization of the C2H2 zinc finger protein Msn2p is regulated by stress and protein kinase A activity. *Genes Dev* **12**, 586-597 (1998).

17 Gietz, R. D. & Sugino, A. New yeast-*Escherichia coli* shuttle vectors constructed with in vitro mutagenized yeast genes lacking six-base pair restriction sites. *Gene* **74**, 527-534 (1988).

18 Pan, X. & Heitman, J. Cyclic AMP-dependent protein kinase regulates pseudohyphal differentiation in Saccharomyces cerevisiae. *Mol Cell Biol* **19**, 4874-4887 (1999).

19 Hill, J. E., Myers, A. M., Koerner, T. J. & Tzagoloff, A. Yeast/E. coli shuttle vectors with multiple unique restriction sites. *Yeast* **2**, 163-167 (1986).

20 Wilson, R. B. & Tatchell, K. SRA5 encodes the low-Km cyclic AMP phosphodiesterase of Saccharomyces cerevisiae. *Mol Cell Biol* **8**, 505-510 (1988).
